# Analyses of Fatal Cases of Kawasaki Disease in Japan Using Vital Statistical Data over 27 Years

**DOI:** 10.2188/jea.13.246

**Published:** 2007-11-30

**Authors:** Shinya Hayasaka, Yosikazu Nakamura, Mayumi Yashiro, Ritei Uehara, Izumi Oki, Morihiro Tajimi, Toshiyuki Ojima, Masaru Terai, Hiroshi Yanagawa

**Affiliations:** 1 Department of Public Health, Jichi Medical School.; 2 Department of Pediatrics, Chiba University School of Medicine.; 3 Saitama Prefectural University.

**Keywords:** case fatality rate, epidemiology, Japan, mortality, mucocutaneous lymph node syndrome

## Abstract

BACKGROUND: Chronological changes in mortality and case fatality rates from Kawasaki disease covering an extended period in Japan are still unknown.

METHODS: We analyzed 679 deaths of patients in Japan whose underlying cause was Kawasaki disease, by using the data of vital statistics between 1972 and 1998.

RESULTS: The male-to-female ratio of the number of deaths was 2.07 and the mean age at death in males was higher. Two unusual increases in the epidemic years, 1982 and 1986, were observed in the chronological changes of the number of deaths. The mortality rate of males was higher than that of females, with a few exceptional years, and the annual mortality rates were high in three epidemic years. The age-specific mortality rate was highest in infants under one year of age. Prefectures with high mortality rate clustered in some regions. The case fatality rate decreased annually, declining to as low as 0.2% among those who were born in 1986 and thereafter; and unusual increases in the case fatality rate affected by these three epidemic years were not repeated.

CONCLUSIONS: The case fatality rate from Kawasaki disease in Japan decreased during the 27 years of observation: improvements in treatment might account for this.

Kawasaki disease (KD) was first described by Dr. Kawasaki in 1967.^1^ In infants and toddlers, the disease is responsible for acute carditis, coronary aneurysms and other cardiac sequelae, which occur in 10 to 20% of the patients.^2^ Sixteen nationwide epidemiologic surveys on KD have been conducted bi-annually since 1970 in Japan. In the 15th survey, approximately 6,500 patients were reported in 1999.^3^

Some researchers have observed mortality and case fatality rates of this disease over the short term.^4^^-^^9^ However, not observed in detail have been the long-term chronological changes of mortality and case fatality rates covering the period that included three epidemic years (1979, 1982, and 1985-1986).^10^^,^^11^ To observe the chronological changes in these rates, we analyzed 679 deaths in patients whose underlying cause was KD by using the data of Japanese vital statistics between 1972 and 1998.

## METHODS

The subjects were all the patients who were born in 1962 and thereafter and succumbed from the sequelae of KD, which is defined by the International Classification of Diseases (ICD), code 695.1 in revised ICD 8 (1972 - 1978), code 446.1 in revised ICD 9 (1979 - 1994), and code M 30.3 in ICD 10 (1995 - 1998). We obtained patients’ date of birth, sex, date of death, and address from Japanese vital statistics; however, information about the immediate cause of death could not be obtained. The few patients who were born before 1961 were excluded from this analysis. These data were officially released for use in this study by the Administrative Management Agency of the Japanese Government.

First, the number of patients and age at death characterized by sex and calendar year were examined. Difference in sex at the means age of death was tested by Student’s t test.

Second, the annual mortality rate per 100,000 children was calculated for both sexes and by age at death and Prefecture. These rates were calculated for patients who were between zero and nine years of age because only a few were 10 years of age or older. The population from the 1985 census was used (it was the medial year in the observed period of time) to calculate the mortality rate for each year (except for the aforementioned medial year).

Third, the expected number of deaths from 1972 through 1998 was calculated by using the mortality rates obtained from the Japanese vital statistics data for each Prefecture. The observed number of deaths was then compared against the expected number derived from a Poisson distribution.

Finally, the case fatality rate characterized by birth year was calculated from data taken from the first to 15th nationwide epidemiologic surveys on KD. The patients reported in these surveys had to satisfy the diagnostic criteria for KD provided by the Japan Kawasaki Disease Research Committee.^12^

Statistical analyses were performed with the SPSS^®^ 10.0J, a statistical program for Windows (SPSS Inc., Chicago, IL, U.S.A.). Values where p < 0.05 were considered to be statistically significant.

## RESULTS

Between 1972 and 1998, 771 deaths of patients were reported in the Japanese vital statistics in which the underlying causes of KD were noted. Of these, 679 (88.1%) who were born in 1962 and thereafter were analyzed. The sex and age distributions of fatal cases are shown in Table 1. The male-to-female ratio was 2.07; and 90% of the patients were under 10 years of age. The mean age at death for males was significantly higher than for females (p < 0.001).

**Table 1.** The number of fatal patients with Kawasaki disease in Japan, 1972-1998, by age and sex.

		Total	Male	Female
		n (%)	n (%)	n (%)
Total		679 (100)	458 (100)	221 (100)
Age at death	0-5 months	136 (20)	82 (18)	54 (24)
	6-11 months	143 (21)	93 (20)	50 (23)
	1-1.4 years	63 (9)	41 (9)	22 (10)
	1.5-1.9	51 (8)	34 (7)	17 (8)
	2	68 (10)	44 (10)	24 (11)
	3	45 (7)	32 (7)	13 (6)
	4	30 (4)	21 (5)	9 (4)
	5-9	74(11)	53 (12)	21 (10)
	10+	69 (10)	58 (13)	11 (5)

Mean ± standard deviation, months*		42.4 ± 59.7	48.3 ± 66.4	30.0 ± 40.3

* Student’s t test, p < 0 .001

**Figure 1.**
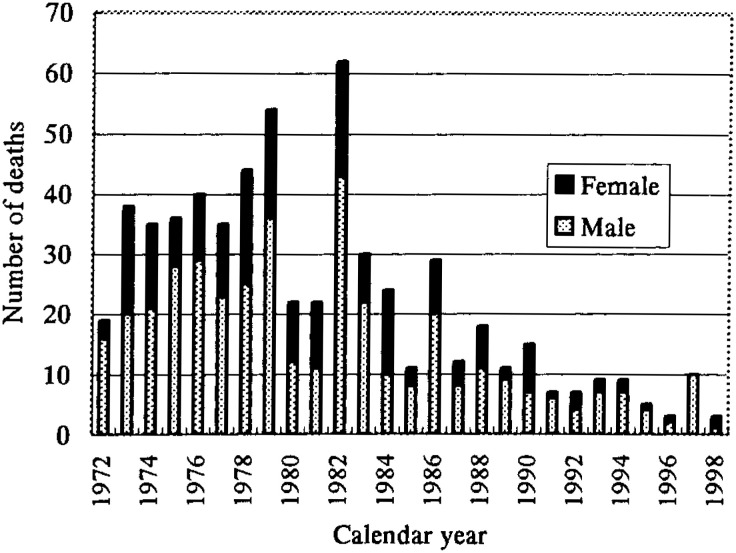
The annual number of deaths of Kawasaki disease in Japan, 1972-1998, by sex.

Figure 1 shows the chronological changes in the number of deaths. The number of deaths in 1982 was 2.8-fold that of the preceding year and a 2.6-fold increase was observed in 1986. These unusual increases occurred in epidemic years.^10^^,^^11^ Another epidemic year was 1979;^10^ however, in the prior year, there was little change. Since 1987, the number of deaths has decreased gradually, being approximately less than 20 per year.

**Figure 2.**
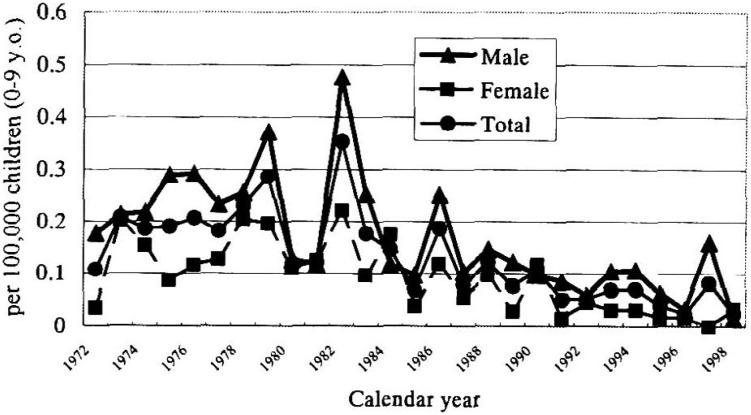
Annual mortality rate of Kawasaki disease in Japan, 1972-1998, by sex.

The annual mortality rate per 100,000 children aged 0-9 years is shown by sex in Figure 2. Throughout the observed period, the overall mortality rates per 100,000 children from zero to nine years of age were 0.17 for males, 0.092 for females, with a male-to-female ratio of 1.84. In almost all years, the mortality rates of males were higher than those of females. In 1979, 1982 and 1986, which were three epidemic years,^10^^,^^11^ the mortality rates were high.

**Figure 3.**
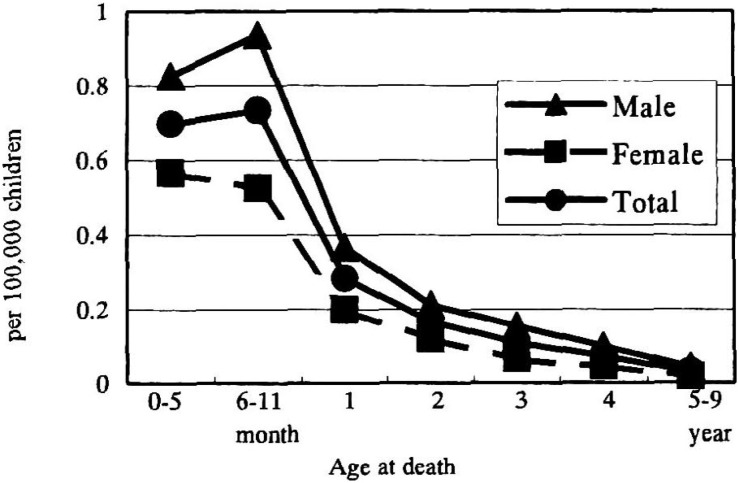
Age-specific mortality rate of Kawasaki disease in Japan, by sex.

The mortality rates per 100,000 children by age at death and sex are shown in Figure 3. Mortality rates were highest among infants under one year of age and decreased with age for both sexes. For all ages at death, the rates for males were higher than for females.

**Figure 4.**
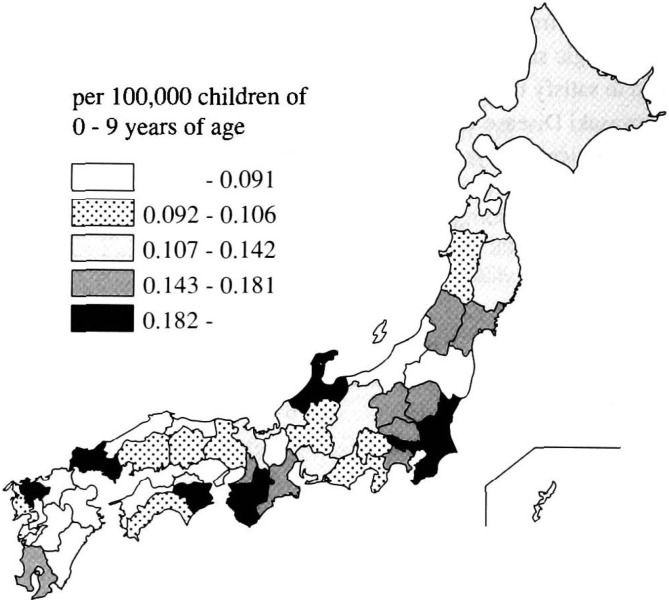
Mortality rate of Kawasaki disease in Japan, 1972-1998, by prefecture.

The mortality rates per 100,000 children by Prefecture are shown in Figure 4. High mortality rates were observed in Nara (0.28), Tokushima (0.24), Ishikawa (0.24), Ibaraki (0.22), and Tokyo (0.21). The Prefectures with a high mortality rate were clustered in the Tokyo metropolitan area and surrounding Prefectures in central Japan. Throughout Japan, the mortality rate per 100,000 children was 0.14. The expected number of deaths for each Prefecture was also calculated. The expected number of deaths from 1972 through 1998 was calculated by multiplying each prefectural population (0-9 years of age) by the mortality rate for all of Japan (i.e., 0.14) to be used as the standard mortality rate. The observed number of deaths was then compared with the expected number by employing a Poisson distribution. As a result, in Tokyo (p < 0.001), Nara (p = 0.01), Ibaraki (p = 0.03) and Chiba (p = 0.03), the observed numbers of deaths were significantly higher than the expected numbers. On the other hand, in Fukushima (p < 0.001), Niigata (p = 0.03), and Okinawa (p = 0.04) the observed numbers of deaths were lower than the expected numbers all with statistical significance.

**Figure 5.**
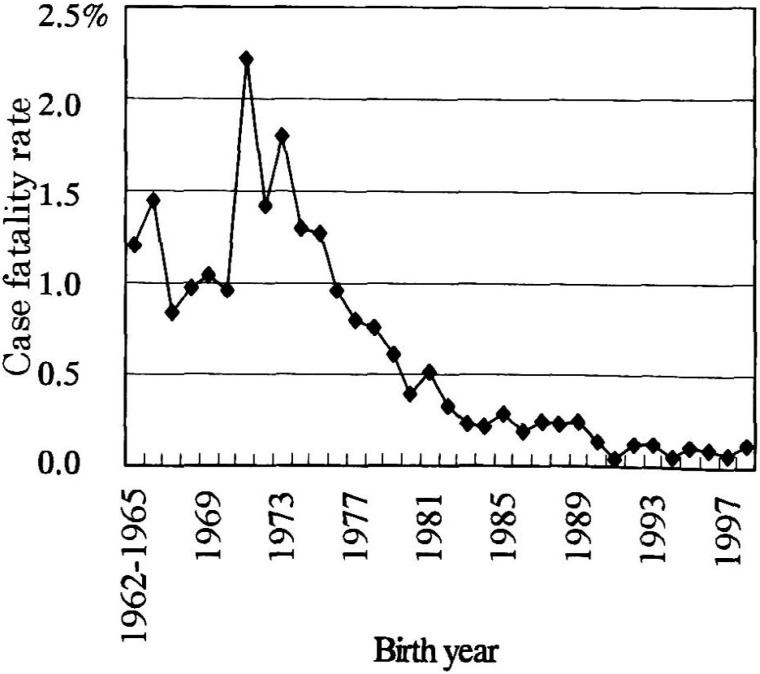
Case fatality rate of Kawasaki disease in Japan by birth cohort.

The case fatality rate by birth year was calculated from the data taken from the first to 15th nationwide epidemiologic surveys on KD, as shown in Figure 5. In these 15 surveys, a total of 153,803 patients with KD were reported through the end of 1998.^3^ Of these, 679 who were born in 1962 and succumbed later were analyzed to obtain the fatality rate. The case fatality rates from 1962 to 1965 were rounded because only a few of the patients who were born in these years were reported. The case fatality rate was highest (2.2%) among those who were born in 1971 and decreased year by year thereafter; but unusual increases in the three epidemic years affected the computations so that no outstanding trends were observed in this period.

## DISCUSSION

We extended the observation period and analyzed 679 deaths, the underlying causes of which was KD, by using the data of vital statistics accumulated in Japan between 1972 and 1998, to observe chronological changes in the mortality and case fatality rates. The results indicate that (1) the male-to-female ratio of the number of deaths was 2.07 and the mean age at death for males was higher than it was for females; (2) two unusual increases in epidemic years, 1982 and 1986, were observed in the chronological changes of the number of deaths; (3) the mortality rate for males was higher than for females, with exceptions in a few years; and the annual mortality rates were high in the three epidemic years; (4) the age-specific mortality rate was highest among infants under one year of age; (5) Prefectures with a high mortality rate clustered in some areas; and (6) the case fatality rate decreased year by year and the three epidemic years did not seem to affect the case fatality rate unduly.

The male-to-female ratio of the number of deaths was 2.07. This result is similar to those of previous studies.^6^^,^^8^^,^^13^^,^^14^ The reason for the difference in the number of deaths by sex may be: (1) the number of male patients with KD was greater than that of females;^3^^-^^9^ and (2) the case fatality rate in males was higher than that in females.^15^

The mean age at death for males was higher than for females. This difference was ascribed to the cardiac sequelae of KD. The presence of cardiac lesions in the acute phase and cardiac sequelae, such as carditis, coronary aneurysms, and myocardial infarction, seriously affects the prognosis of the disease. Starting with the 8th nationwide survey, an item about the cardiac sequelae of KD was included in the questionnaire.^16^ The proportion of cardiac sequelae in males was greater than it was in females.^6^^,^^7^^,^^16^ The sex-related difference in the mean age at death was explained by the difference in the sex-related incidence of cardiac sequelae.

Two unusual increases were observed in the chronological changes in the number of deaths in 1982 and 1986. Case fatality rates had hardly changed in these years when compared with the preceding years.^15^ On the other hand, 1982 and 1986 were epidemic years throughout Japan and many patients with KD were reported then.^7^ These unusual increases in the number of deaths may be explained by an increase in the number of patients with KD.

The mortality rates of males were higher than those of females (except for some years). This rate expresses the number of deaths in a particular population during a given period.^17^ The mortality rate is associated with the incidence and case fatality rates. In each year, the incidence rate among males was higher than that in females.^6^^-^^8^ In addition, the case fatality rate among males was higher than among females in all age groups.^15^ Therefore, the mortality rate of males was higher than females in almost every year.

Annual mortality rates were high in the three epidemic years of 1979, 1982 and 1986. Many patients with KD were reported in these years.^7^ When compared with the preceding years,^15^ the case fatality rates had hardly changed in these three years. The high mortality rates in these epidemic years may be explained by the increase in the number of patients with KD.

It was observed that the mortality rate, highest among infants under one year of age, decreased with age. The age-specific incidence rates among infants under one year of age and in those one year old were similar for both sexes in each year.^18^ The incidence rate among children under and equal to one year of age was the highest but the rate decreased with age.^3^^-^^6^^,^^8^^,^^9^ In addition, the case fatality rates among infants under one year of age were highest and decreased with age in both sexes.^15^ For these reasons, the mortality rates were also highest in children under one year of age and decreased as they grew older.

Prefectures with a high mortality rate were clustered in some areas. Previous studies had found that those Prefectures with high incidence rates were concentrated around the Tokyo Metropolitan area and the surrounding Prefectures in central and western Japan.^6^^,^^8^^,^^9^ The local high mortality may be affected by the clustering of incidence rates. Another study had noted that the most recent epidemic started in Tokyo in 1985 and the disease spread throughout Japan within six months.^5^ This concentration of mortality and incidence rates and migration of the epidemic areas may suggest that the etiology of the disease is infectious although that is yet to be proven.

The case fatality rate decreased annually and the unusual increases in this rare that one might have expected from the three epidemic years were not reflected clearly in this study. Among those patients born in the latter half of the 1970s and thereafter, the case fatality rate decreased rapidly in subsequent years. A previous study reported yearly trends in types of treatment.^7^ In the latter half of the 1970s, the proportion of those treated with steroidal agents decreased, and almost all the patients were treated with aspirin. The proportion of patients who were treated with immunoglobulin was only 2.5% in 1982 but increased sharply to 33.5% in 1985, 54.8% in 1988, and 81.8% in 1992.^7^^,^^16^ This decrease in case fatality rate was probably associated with the change in the type of treatment.

In conclusion, it has been shown the male-to-female ratio is high for the number of deaths and the mortality rates for males is higher than for females; high mortality rates clustered in some areas; and annual mortality rates were high in the three epidemic years. However, the case fatality rate decreased every year and unusual increases that occurred during these three epidemic years were not evidenced.
